# Editorial: Advances in vaginal microbiome and metabolite research: genetics, evolution, and clinical perspectives

**DOI:** 10.3389/fcimb.2025.1755233

**Published:** 2025-12-15

**Authors:** António Machado, Claudio Foschi, Qinping Liao, Bingbing Xiao, Antonella Marangoni

**Affiliations:** 1 Universidade dos Açores, Faculdade de Ciências e Tecnologia, Departamento de Biologia, Centro de Biotecnologia dos Açores (CBA), Ponta Delgada, Portugal; 2 Universidad San Francisco de Quito (USFQ), Colegio de Ciencias Biológicas y Ambientales (COCIBA), Instituto de Microbiología, Laboratorio de Bacteriología, Quito, Ecuador; 3 Microbiology, Department of Experimental, Diagnostic and Specialty Medicine, University of Bologna, Bologna, Italy; 4 Microbiology Unit, Istituto di Ricovero e Cura a Carattere Scientifico (IRCCS) Azienda Ospedaliero-Universitaria of Bologna, Bologna, Italy; 5 Beijing Tsinghua Changgung Hospital, School of Clinical Medicine, Tsinghua University, Beijing, China; 6 Department of Obstetrics and Gynecology, Peking University First Hospital, Beijing, China

**Keywords:** biofilms, cervical neoplasia, human papillomavirus, metabolomics, microbial dysbiosis, microbiota-metabolite interactions, mucosal immunity, vaginal microbiome

The vaginal microbiome is a dynamic and highly specialized environment in which microbial communities, metabolites, mucosal immunity, hormonal signals, and host factors interact continuously. A stable microbiota dominated by *Lactobacillus* species has long been considered a hallmark of vaginal health, largely due to the production of lactic acid, maintenance of a protective acidic pH, block or displacement of opportunistic pathogens, and the modulation of inflammatory responses (Ceccarani et al., 2019; MaChado et al., 2022; Rodríguez-Arias et al., 2022). However, recent technological advances in high-resolution sequencing, metabolomics, immunology, and evolutionary genomics have revealed that this balance is far more intricate than previously appreciated. Subtle shifts in microbial composition or function can influence susceptibility to infections, viral persistence, progression of cervical neoplasia, treatment outcomes, and pregnancy-related complications (MaChado et al., 2025).

**Figure 1:**
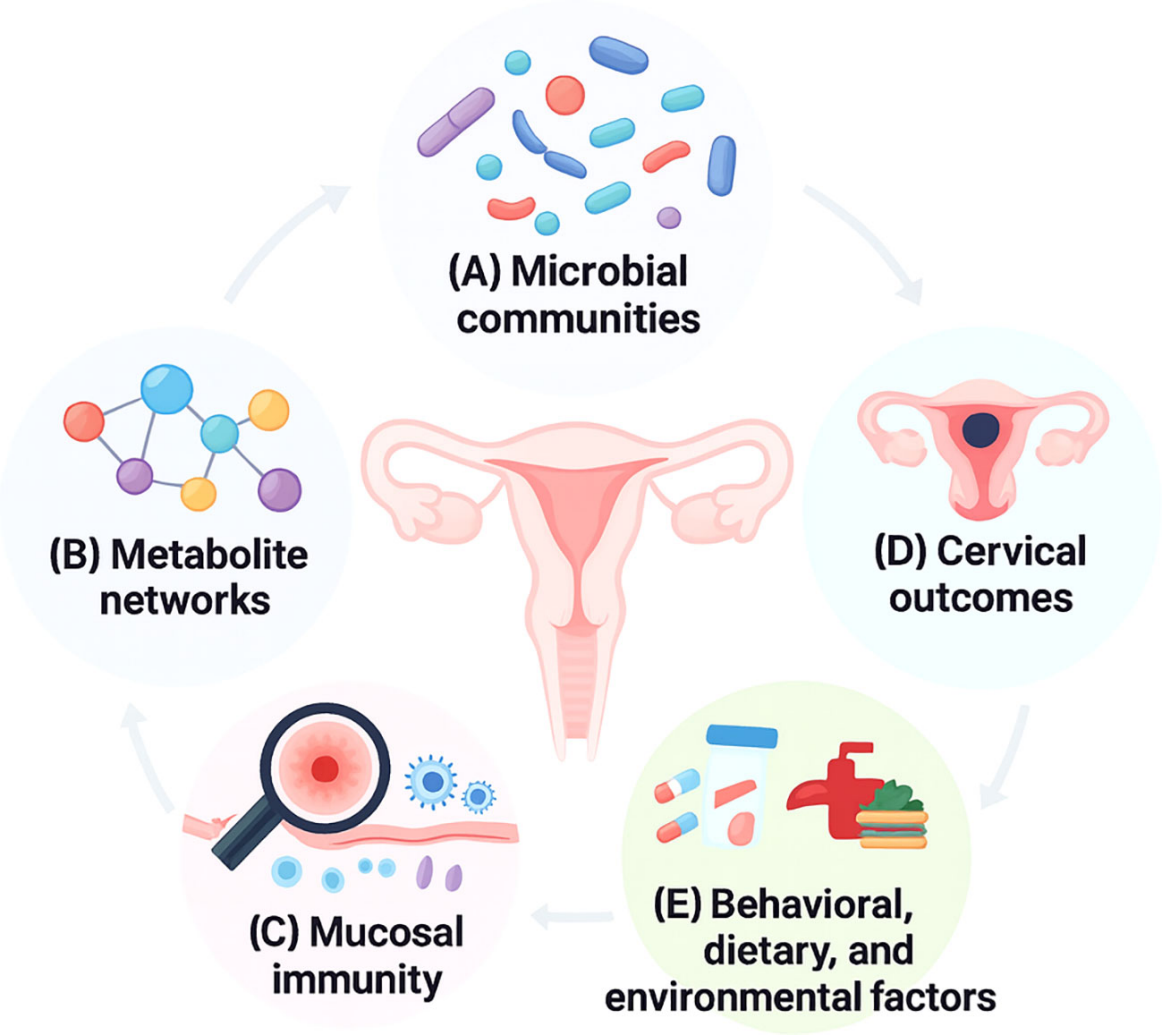
Integrated conceptual model of the vaginal ecosystem: microbiota-metabolite-immune-clinical interactions. This diagram illustrates the interconnected components of the vaginal ecosystem and their roles in reproductive health and disease: **(A)** Microbial communities reflect protective *Lactobacillus*-dominated CSTs or dysbiotic anaerobic profiles; **(B)** Metabolite networks represent functional outputs influencing pH, inflammation, and viral persistence; **(C)** Mucosal immunity encompasses cytokine signaling, epithelial responses, and inflammation linked to microbial states and infections; **(D)** Cervical outcomes include HPV acquisition, persistence, lesion progression, and cancer risk shaped by microbiota-metabolite-immune interactions; and **(E)** Behavioral, dietary, and environmental factors modulate microbial composition, metabolic activity, and long-term vaginal health.

The Research Topic “*Advances in Vaginal Microbiome and Metabolite Research: Genetics, Evolution, and Clinical Perspectives*” was conceptualized to assemble contributions reflecting this multidimensional field (see **Figure 1**). The fifteen articles included offer complementary perspectives that collectively advance our understanding of how microbial ecology, metabolic pathways, immune activation, pathogen evolution, and clinical factors converge within the reproductive tract. Taken together, these studies illustrate both the opportunities and remaining challenges for translating vaginal microbiome research into predictive, preventive, and personalized medicine.

A major theme emerging from this Research Topic is the close interplay between microbiota composition, metabolic signatures, and viral dynamics, especially regarding human papillomavirus (HPV). Several authors apply untargeted and targeted metabolomics to identify metabolic shifts associated with HPV persistence and treatment response. Li et al. used combined untargeted and targeted metabolomics to characterize vaginal lavage samples from women with persistent HPV infection treated with a traditional Chinese herbal formulation, Jiawei Ermiao Granule, revealing a panel of small-molecule biomarkers and highlighting amino sugar and nucleotide sugar metabolism, starch and sucrose metabolism, and related pathways as potentially relevant axes of therapeutic action. Their work illustrates how metabolomics can be leveraged to elucidate mechanisms of action for multi-component interventions and to explore the microenvironmental changes associated with viral clearance.

Focusing on structural changes in microbial communities, Liang et al. evaluated the vaginal microbiota in women with persistent HPV infection before and after cervical conization and in women who cleared HPV post-surgery. They showed that *Lactobacillus* remains a predominant genus, but that persistent HPV infection after conization is associated with increased microbial diversity, higher abundance of bacterial vaginosis (BV)–associated anaerobes, and signatures of anaerobic and biofilm-forming phenotypes. *Gardnerella*, *Atopobium*, and other anaerobes were enriched in women with persistent HPV, with clear evidence of co-occurrence patterns and negative correlations between *Lactobacillus* and several pathogenic taxa. In addition, these authors postulated that biofilm formation may play a significant role in the persistence of HPV infection post-surgery proposing that the role of *Gardnerella* in HPV-infected state warrants further study. These findings reinforce the notion that surgical treatment of cervical lesions does not necessarily restore a protective microbial ecosystem and that persistent dysbiosis may contribute to viral persistence.

At a more advanced stage of disease, Zhai et al. integrated microbiome and metabolome profiling of cervical swabs from women with healthy cervices, high-grade squamous intraepithelial lesions (HSIL), and cervical cancer (CC). Their analysis revealed increased microbial diversity, reduced *Lactobacillus* abundance, and major shifts in microbial composition in CC compared with healthy controls and HSIL. Metabolomics identified elevated levels of the inflammatory mediator prostaglandin E2 in CC samples and, through random forest models, a combination of key taxa (such as *Porphyromonas*) and metabolites (including cellopentaose and prostaglandin E2) with high diagnostic value for CC. The strong correlation between specific bacteria and inflammatory metabolites supports a model in which microbial-induced inflammation contributes to the aggravation of cervical neoplasia.

Across these studies, a recurrent pattern emerges: the vaginal microbiota and the local metabolome are not passive passengers during HPV infection and cervical carcinogenesis but active determinants of risk and disease trajectory. The work by Li et al., Liang et al., and Zhai et al. collectively demonstrates that metabolomics, when combined with high-resolution microbial profiling, can reveal both mechanistic insights and promising biomarker candidates for monitoring persistent HPV infection and lesion progression.

Another important theme in this Research Topic is the immunological landscape of the vaginal mucosa and its relationship with dysbiosis and sexually transmitted infections. Young et al. characterized cytokine concentrations and T cell subsets in cervicovaginal lavage specimens from women with and without BV and *Trichomonas vaginalis* (TV) infection in Atlanta, Georgia. These authors found that the concentrations of the majority of measured cytokines and most CD4+ T cell subsets differed by infection status. In multivariable models, TV infection was associated with a higher T cell score, reflecting increased vaginal mucosal T cell concentrations, while BV was not associated with a higher composite T cell score. These findings provide a plausible mechanistic link between TV infection and increased HIV risk, highlighting how specific vaginal infections may shape the immunological microenvironment in ways that promote susceptibility to other pathogens.

Lifestyle and environmental factors further shape the vaginal ecosystem, as illustrated by the study of Djusse et al., who explored the relationship between dietary habits, vaginal microbiota, and vaginal metabolome in a cohort of young, non-pregnant women. By combining *16S* rRNA gene profiling, ^1^H-NMR-based metabolomics, and detailed dietary assessment, they found that increased intake of animal proteins, particularly from red and processed meat, was positively associated with a dysbiotic Community State Type (CST) IV, whereas alcohol consumption correlated with higher levels of *Gardnerella* and *Ureaplasma*. In contrast, α-linolenic acid intake was inversely associated with CST III, dominated by *Lactobacillus iners*, and positively related to *L. crispatus*, which in turn was linked to beneficial metabolites such as 4-hydroxyphenyllactate and several amino acids. Total carbohydrates, vegetable proteins, fiber, and starch were negatively correlated with *Gardnerella* abundance. These findings indicate that dietary composition may influence the vaginal microbiota and metabolome in ways that either promote or protect against dysbiosis, opening new avenues for preventive strategies.

Furthermore, Cui et al. provided a comprehensive review of the interrelated nature of vaginal microecology, HPV infection, and cervical lesion progression. Their review highlights the central role of *Lactobacillus*-dominated communities in maintaining an acidic environment and robust mucosal defenses through lactic acid, hydrogen peroxide, and bacteriocin production. Conversely, dysbiosis, especially dominance of CST IV communities enriched in *Gardnerella*, *Prevotella*, and *Atopobium*, can increase susceptibility to high-risk HPV (HR-HPV), facilitate viral persistence, and promote cervical lesion development. A key contribution of their review is the articulation of a bidirectional model in which HPV infection itself can destabilize the microbiota, creating a feedback loop between inflammation, elevated vaginal pH, immune dysregulation, and microbial imbalance. By analyzing clinical, microbial, and immunological data, Cui et al. underscore the need for integrated microbial–immune biomarkers for early diagnosis and prevention.

At the clinical–diagnostic interface, Peng et al. examined how measurable microecological indicators correlate with HR-HPV infection. In a large cross-sectional cohort, they demonstrated that HR-HPV positivity is significantly associated with higher vaginal pH, increased sialidase and leukocyte esterase activity, reduced hydrogen peroxide production, and markedly lower *Lactobacillus* abundance. Their ROC curve analysis (AUC 0.701) indicates that routine microecological parameters, already used in many gynecological clinics, offer moderate predictive value for HR-HPV infection. The study provides empirical support for incorporating simple microecological indicators into HPV risk stratification. The age distribution and subtype patterns reported by Peng et al. also reinforce epidemiological links between dysbiosis, reproductive age, and HR-HPV acquisition.

Adding another layer of complexity, Kawasaki et al. studied how menopausal status modulates the cervical microenvironment across the spectrum of cervical intraepithelial neoplasia and squamous cell carcinoma. Their integrative analysis, encompassing the cervical microbiome, metabolome, cytokines, and miRNA expression, revealed age-specific microbial and metabolic signatures. In younger women with squamous cell carcinoma (SCC), *Atopobium*, *Gardnerella*, and *Adlercreutzia* correlated positively with metabolites such as xanthine and nicotinic acid, while older women exhibited a distinct enrichment of *Peptoniphilus*, *Porphyromonas*, and *Fusobacterium*. High expression of RANTES, miR-20b-5p, and miR-155-5p was linked to altered metabolite profiles, suggesting microbe–metabolite–immune networks that differ by menopausal status. Kawasaki et al. thus present strong evidence that menopausal hormonal transition reshapes the cervical microenvironment in ways that may influence cancer progression pathways.

Large-scale epidemiological perspectives were provided by Lin et al., who analyzed microecological and infection patterns in more than 27,000 gynecologic outpatients. Their findings show profound age-related shifts: postmenopausal women exhibited significantly higher rates of BV and aerobic vaginitis, lower prevalence of vulvovaginal candidiasis (VVC), increased inflammatory markers, and distinct metabolomic profiles such as elevated bile-acid conjugates. These results illustrate how estrogen decline influences microbial diversity, epithelial atrophy, and immune tone—factors that collectively modulate susceptibility to dysbiosis and infection. By integrating clinical epidemiology with metabolomic data, Lin et al. demonstrated that menopausal status should be considered a major biological determinant of vaginal ecosystem configuration and infection risk.

In addition, Buchta et al. explored how microbial community stability and *Lactobacillus* species dynamics relate to chronic vulvovaginal discomfort (CVD), a condition frequently linked to underlying dysbiosis. These authors found that while CST III dominated many patient groups, CST stability differed markedly between CVD patients and healthy controls. Notably, *L. iners* and *L. gasseri* exhibited opposite abundance trends depending on clinical status, suggesting that the ecological roles of these species depend heavily on community context. CVD patients showed a higher prevalence of unstable CSTs, more Gram-positive cocci, and a distinctive pattern of CST transitions, indicating long-term ecological perturbation. These insights deepen our understanding of CST instability as both a contributor to and consequence of chronic symptoms.

Shifting from host-pathogen interaction to microbial evolution and epidemiological surveillance, Shabayek et al. conducted a detailed analysis of CRISPR-Cas systems and prophage content in colonizing Group B *Streptococcus* (GBS) from healthy Egyptian women, providing an evolutionary and genomic lens on maternal carriage. Their work revealed a high prevalence of CRISPR arrays and prophages across isolates, with remarkable congruence between MLST sequence types, CRISPR1 spacer profiles, and prophage repertoires. The identification of region-specific African lineages emphasizes the importance of population-level genomic monitoring and highlights CRISPR typing as a low-cost, high-discriminatory alternative to MLST in resource-limited settings. These findings are particularly timely given the ongoing development of maternal GBS vaccines and cost-effective typing tools such as CRISPR profiling will be essential for post-vaccine molecular surveillance.

Complementing this genomic perspective, Mai et al. realized a comprehensive analysis of GBS isolates from pregnant women and neonates in Haikou, China, integrating antibiotic susceptibility, virulence genes, serotypes, sequence types, and whole-genome sequencing. Their dataset, encompassing 138 isolates, revealed a striking dominance of tetracycline (89.1%) and clindamycin (55.1%) resistance, with widespread presence of key determinants such as *mreA*, *ermB*, and *tetM*. The identification of ST862 as a prevalent and potentially zoonotic lineage underscores the need for One Health–oriented approaches in GBS control. Virulence factors were ubiquitous, with all strains carrying *cfb* and *cylE*, emphasizing the pathogenic potential within colonizing populations. The coexistence of multiple serotypes and clonal complexes within this tropical region further illustrates the genetic diversity of GBS and highlights the importance of regional data for vaccine formulation and antimicrobial stewardship.

The intersection between antibiotic exposure, lifestyle factors, and microbial ecology was explored by Castellano et al., who examined the vaginal resistome of reproductive-age women and its association with microbial community structure and behavioral traits. They detected high prevalence of macrolide- and tetracycline-resistance genes, particularly *erm(F)*, *tet(M)*, and *erm(B)*, alongside strong positive associations between antibiotic resistance genes (ARG) burden and CST IV communities enriched in *Gardnerella* and *Prevotella*. In contrast, *Lactobacillus*-dominated CSTs were inversely correlated with ARG presence. Notably, smoking, higher body mass index (BMI), recent antibiotic use, and presence of *Candida* spp. were linked to increased ARGs detection, whereas oral contraceptive use and higher dietary quality scores appeared protective. This study provides important public health insights by showing that the vaginal microbiota acts not only as a biological ecosystem but also as a behavioral and environmental sensor, with antibiotic resistance shaped by modifiable exposures.

In the context of non-viral sexually transmitted infections, Jun et al. investigated mixed vaginal infections among women infected with *Trichomonas vaginalis* (TV), comparing traditional microscopy with metagenomic sequencing for microbial detection and CST classification. Their findings demonstrate the substantial diagnostic limitations of wet-mount microscopy, which failed to identify many co-infections detected through metagenomics. TV-positive women frequently harbored mixed communities characterized by CST IV profiles and an increased abundance of anaerobic, BV-associated species. The authors highlight how TV co-infection disrupts microbiota stability, amplifies inflammatory responses, and complicates clinical management in settings where drug resistance and recurrence are growing concerns. Their comparison underscores the value of molecular methods for detecting mixed infections that might otherwise remain unrecognized yet have important implications for symptom persistence and treatment failure.

Finally, Cancio-Suárez et al. examined the cervical microbiota of women across HPV-negative, low-grade squamous intraepithelial lesion (LSIL), and high-grade squamous intraepithelial lesion (HSIL) diagnostic categories, revealing microbial and functional alterations associated with progression toward high-grade lesions. Their findings indicate that HSIL samples exhibit higher microbial diversity and an enrichment of genera such as *Parvimonas*, *Fastidiosipila*, and *Pseudomonas*, accompanied by functional signatures involving glycine, serine, threonine, and sulfur metabolism. These metabolic pathways may contribute to microenvironmental changes that facilitate neoplastic progression. The study reinforces prior evidence that cervical and vaginal communities, while anatomically contiguous, exhibit site-specific ecological patterns that are clinically relevant. By integrating microbial composition with predicted functional shifts, Cancio-Suárez et al. highlight the potential of microbiome-informed biomarkers to identify women at elevated risk of HSIL development.

In conclusion, the fifteen studies included in this Research Topic collectively advance our understanding of the vaginal microbiome by integrating microbial ecology, metabolomics, mucosal immunology, evolutionary genetics, and clinical perspectives. Together, they underscore the importance of viewing the vaginal environment as a cohesive ecosystem in which microbial composition, functional pathways, immune responses, and host factors intersect to influence reproductive health. Despite the substantial progress reflected in these contributions, important knowledge gaps remain. Longitudinal studies are needed to establish causal relationships, functional analyses to elucidate microbial mechanisms, and translational efforts to integrate multi-omics data into clinical practice. The diversity of approaches represented here illustrates the value of interdisciplinary collaboration and sets the stage for future advances that may meaningfully enhance diagnostic precision, therapeutic efficacy, and reproductive outcomes worldwide.
